# Star fruit extract and *C*-glycosylated flavonoid components have potential to prevent air pollutant-induced skin inflammation and premature aging

**DOI:** 10.1007/s13659-022-00336-1

**Published:** 2022-04-01

**Authors:** Ping Wu, Hiroyasu Iwahashi, Hai-Hui Xie, Ying Wang, Yan-Yang Zhou, Akinori Kiso, Yoshihito Kawashima, Xiao-Yi Wei

**Affiliations:** 1 Key Laboratory of Plant Resources Conservation and Sustainable Utilization/Guangdong Provincial Key Laboratory of Digital Botanical Garden and Public Science, South China Botanical Garden, Chinese Academy of Sciences, Xingke Road 723, Tianhe District, Guangzhou, 510650 People’s Republic of China; 2 Research Center, Maruzen Pharmaceuticals Co. Ltd., 1089-8 Sagata, Shin-ichi-Cho, Fukuyama-City, Hiroshima 729-3102 Japan

**Keywords:** Star fruit, Carambolaside P, Air pollution, Protein carbonylation, Skin inflammation, Premature skin aging

## Abstract

Air pollution adversely affects skin, leading to skin inflammation and premature skin aging. Plant derived antioxidant compounds have been considered to be promising in discovery of effective agents for the protection of skin from the damage by air pollutants. Our previous studies demonstrated that *Averrhoa carambola* fruit (known as star fruit) is rich in flavonoid *C*-glycosides with unique structures and potent antioxidant activity. Thus, the star fruit extract (SFE) and main flavonoid *C*-glycoside components, carambolasides I, J, and P (**1**–**3**), carambolaflavone B (**4**), and isovitexin 2″-*O*-α-l-rhamnoside (**5**), were investigated for the activity against air pollutant stress in human epidermis. As a result, SFE and compounds **1**–**5** exhibited significant inhibitory activity against protein carbonylation in oxidative-stressed stratum corneum with the best activity being shown by compound **3**. SFE and compounds **2**–**5** were also active against engine exhaust-induced protein carbonylation in stratum corneum. When further evaluated, SFE and compound **3** significantly inhibited gene expression of the key inflammation mediators IL-1α and COX-2 in PM-stressed keratinocytes. The results indicated that SFE and the flavonoid *C*-glycosides are potentially effective against air pollutant-induced skin inflammation and premature aging.

**Graphical Abstract:**

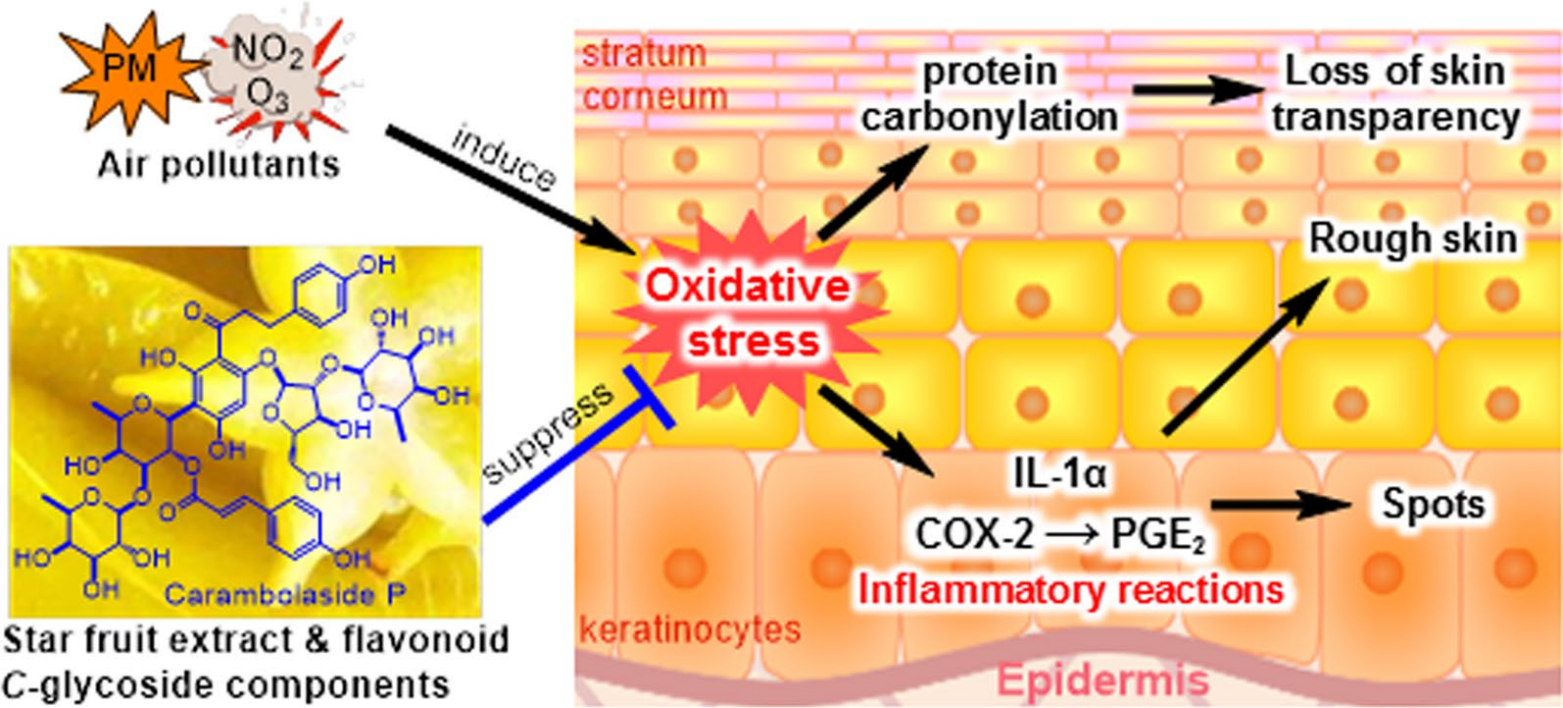

**Supplementary Information:**

The online version contains supplementary material available at 10.1007/s13659-022-00336-1.

## Introduction

Air pollution is a serious threat to public health and life quality [1]. Pollutants in air pollution of major health concern are mainly of anthropogenic origin and include particulate matter (PM) and gaseous ozone, nitrogen oxides, and sulfur oxides. Numerous epidemiological studies have identified that both long- and short-term exposure to airborne pollution, including exposure to both PM and gaseous pollutants increases respiratory and cardiovascular morbidity [1, 2]. Besides, air pollution is also correlated with the progression of inflammatory skin diseases such as atopic dermatitis, acne, psoriasis and allergic reactions, in particular, high levels of air pollutants, including polycyclic aromatic hydrocarbons, heavy metals and oxides contribute to premature skin aging [3]. A study examining 400 German women aged 70–80 years, who resided in urban areas close to highways or country environments for 24 years, showed that air pollution increased skin pigmentation and led to more wrinkles on the skin [4].

The epidermis is the outermost protective barrier of the skin and protects the body from the external environment. The stratum corneum (SC) and epidermal keratinocytes, which constitute the epidermis, are the first sites to encounter air pollutants and are thought to be the most adversely affected. Keratinocytes have been reported to synthesize and secrete pro-inflammatory cytokines, such as interleukin (IL)-1, IL-6, IL-8, tumor necrosis factor (TNF)‐α, and prostaglandin (PG) E_2_, caused by air pollutants [5–9]. In vitro studies have suggested that the PM-induced inflammatory responses are mediated by intracellular reactive oxygen species (ROS) produced via the aryl hydrocarbon receptor (AhR) pathway. ROS are able to activate nuclear factor (NF)-κB and activator protein 1 (AP-1), which can promote the release of IL-1α and PGE_2_ [9]. IL-1α is positively correlated with symptom exacerbation and disease progression in psoriasis, atopic dermatitis, neutrophilic dermatoses, skin phototoxicity, and skin cancer [10]. PGE_2_, which is synthesized by cyclooxygenase 2 (COX-2), also plays a major role in inflammation, edema, keratinocyte proliferation, epidermal hyperplasia, and generation of a pro-oxidant state leading to oxidative DNA damage [11]. Thus, the progression of inflammatory skin diseases due to air pollution is thought to be caused by IL-1α and PGE_2_ and chronic inflammation accelerates aging [12].

Other studies have suggested that premature skin aging is also caused by protein carbonylation of SC, which, known to be mediated by aldehydes generated from lipid oxidation, changes the quality of the skin appearance by altering the fibrous structure of keratin and decreasing the light transmission of SC [13]. Carbonylated protein in the SC is also involved in skin dryness by decreasing skin water content [14, 15]. Skin dryness itself causes SC carbonylation and secretion of various inflammatory factors, including IL-1α, from keratinocytes, leading to epidermal inflammation [16]. Furthermore, these inflammatory conditions may contribute to wrinkle formation by altering the dermal matrix through increased secretion of MMP-1 from dermal fibroblasts.

Based on these studies, it is clear that air pollutant-induced skin diseases and premature skin aging are both medicated by or associated with ROS. In recent years, the theory of aging due to such oxidation and inflammation has been proposed [17]. Accordingly, plant phenolic constituents have attracted attention in discovery of agents for prevention and treatment of air pollutant-induced skin disorders due to their antioxidant capacity [18]. Many extracts and phenolic compounds from plants, such as cocoa, green tea, pomegranate, have been shown to have antioxidant and anti-inflammatory effects on PM-exposed cells and suggested that plant derived phenolic compounds are promising as effective additives for development of antipollution cosmetic products on human skin [18].

**Fig. 1:**
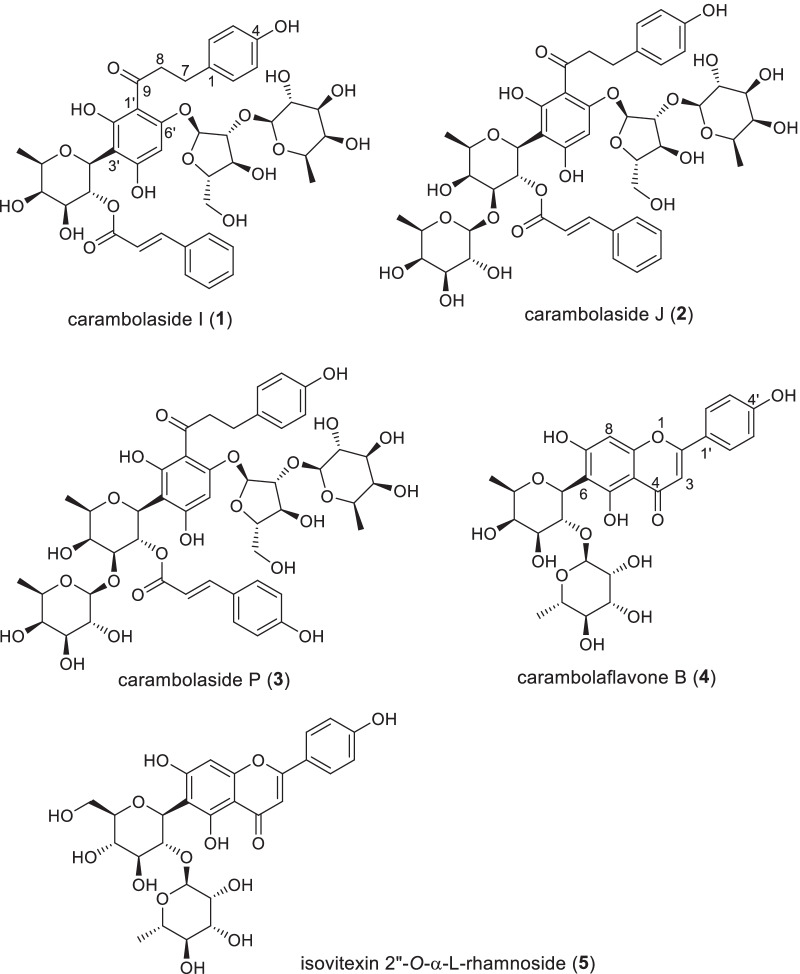
Structures of the main flavonoid *C*-glycosides isolated from star fruit

*Averrhoa carambola* L. (Oxalidaceae), a tree native to tropical Southeast Asia, is widely cultivated in tropical and subtropical regions for its edible fruit, commonly known as star fruit. Our previous phytochemical investigations of this fruit led to the isolation of 17 flavonoid *C*-glycosides with antioxidant activity, including 15 new dihydrochalcones featuring a 3′-*C*-*β*-d-fucosylated phloretin moiety [19–21]. In the present investigation, the star fruit extract (SFE) and main flavonoid *C*-glycosides, carambolasides I, J, and P (**1**–**3**), carambolaflavone B (**4**), and isovitexin 2″-*O*-α-l-rhamnoside (**5**) (Fig. 1), were evaluated for the inhibitory activity against NaClO- and gasoline engine exhaust gas (GEEG)-induced protein carbonylation in stratum corneum (SC) and PM-induced IL-1α and COX-2 gene expression in normal human epidermal keratinocytes (NHEKs). Here are presented details of the investigation.

## Results

### UPLC-HRESIMS analysis of SFE

Before activity evaluations, the prepared SFE was subjected to UPLC-HRESIMS analysis. By interpretation of MS/MS data of the UPLC peaks (Fig. S1 and S2, Supplementary Information) as well as by comparison of the data with previously reported values [19–21], eight flavonoid *C*-glycosides were detected in SFE (Additional file 1: Table S1). The presence of compounds **1**–**3** and **5** was confirmed by comparison with the authentic compound samples obtained in our previous studies [19–21], of which compound **3** was shown to be the most abundant flavonoid *C*-glycoside in SFE by the UV (280 nm)-detected UPLC (Additional file 1: Fig. S1).

### Inhibition of NaClO-induced protein carbonylation in SC

**Fig. 2:**
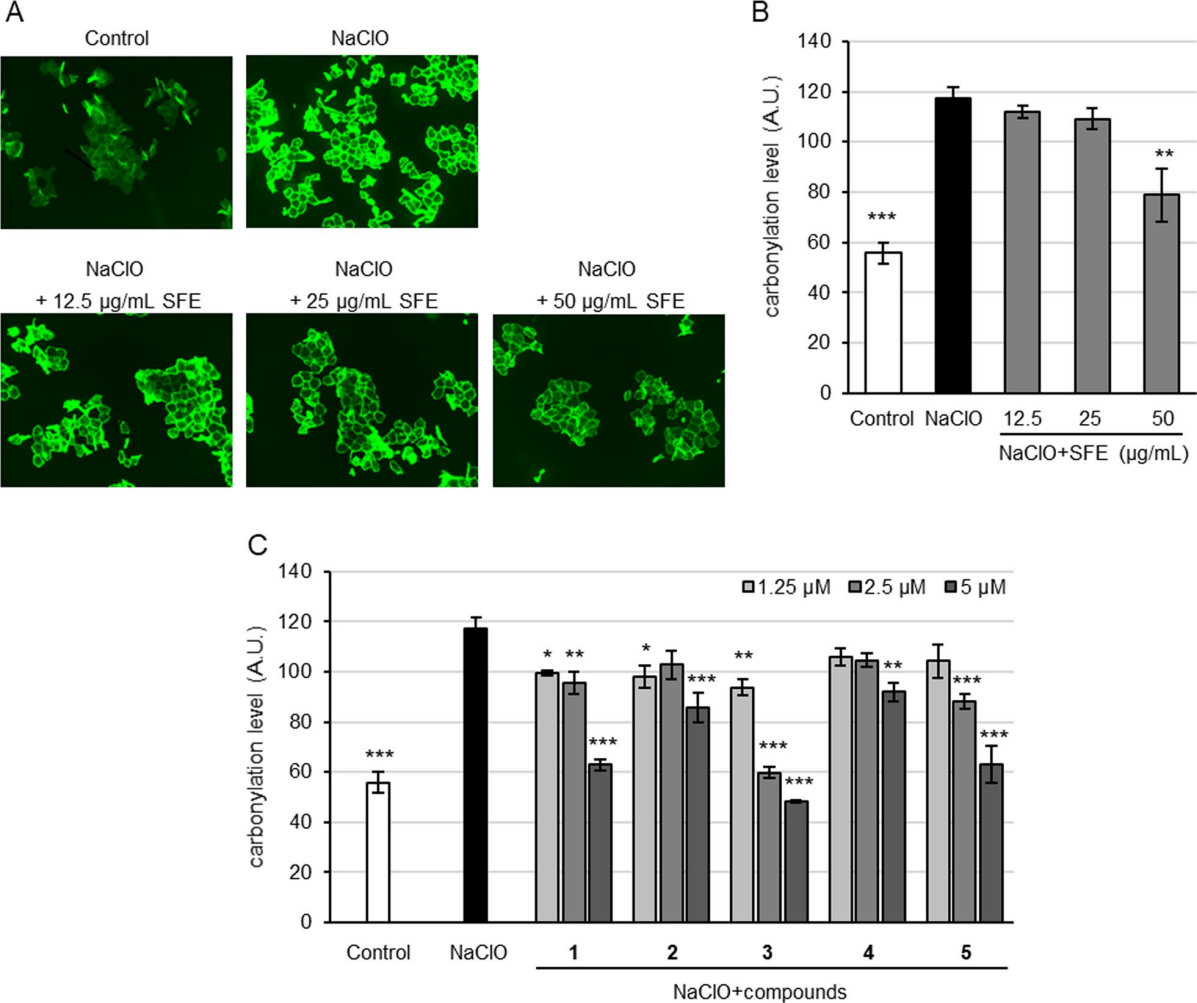
Inhibitory activity of SFE and compounds **1**–**5** against NaClO-induced protein carbonylation in SC. Tape-stripped SC was treated with SFE or components in the absence or presence of NaClO for 16 h at 37 °C. Protein carbonylation in SC were detected by 5-FTSC. Inhibitory activity of SFE (**A**,** B**) and compounds **1**–**5** (**C**) against NaClO-induced protein carbonylation in SC and typical fluorescence image of protein carbonylation (**A**). Bars in B and C represent mean ± S.E., n = 3; *p < 0.05, **p < 0.01, ***p < 0.001 vs. NaClO-treated control.

The evaluation was carried out using the method reported by Iwai et al.^14)^ As a result, SFE was shown to significantly decrease the level of protein carbonylation in NaClO-stressed SC at the dose of 50 μg/mL (Fig. 2A, B). Among compounds **1**–**5**, carambolasides I (**1**) and P (**3**) and isovitexin 2″-*O*-α-d-rhamnoside (**5**) were indicated to inhibit NaClO-induced protein carbonylation in a dose-dependent manner and the best activity was found to be compound **3** (Fig. 2C; Additional file 1: Table S2). The results showed that SFE and the *C*-glycosylated flavonoid components were effective against ROS-induced protein carbonylation in SC.

### Inhibition of GEEG-induced protein carbonylation in SC

**Fig. 3:**
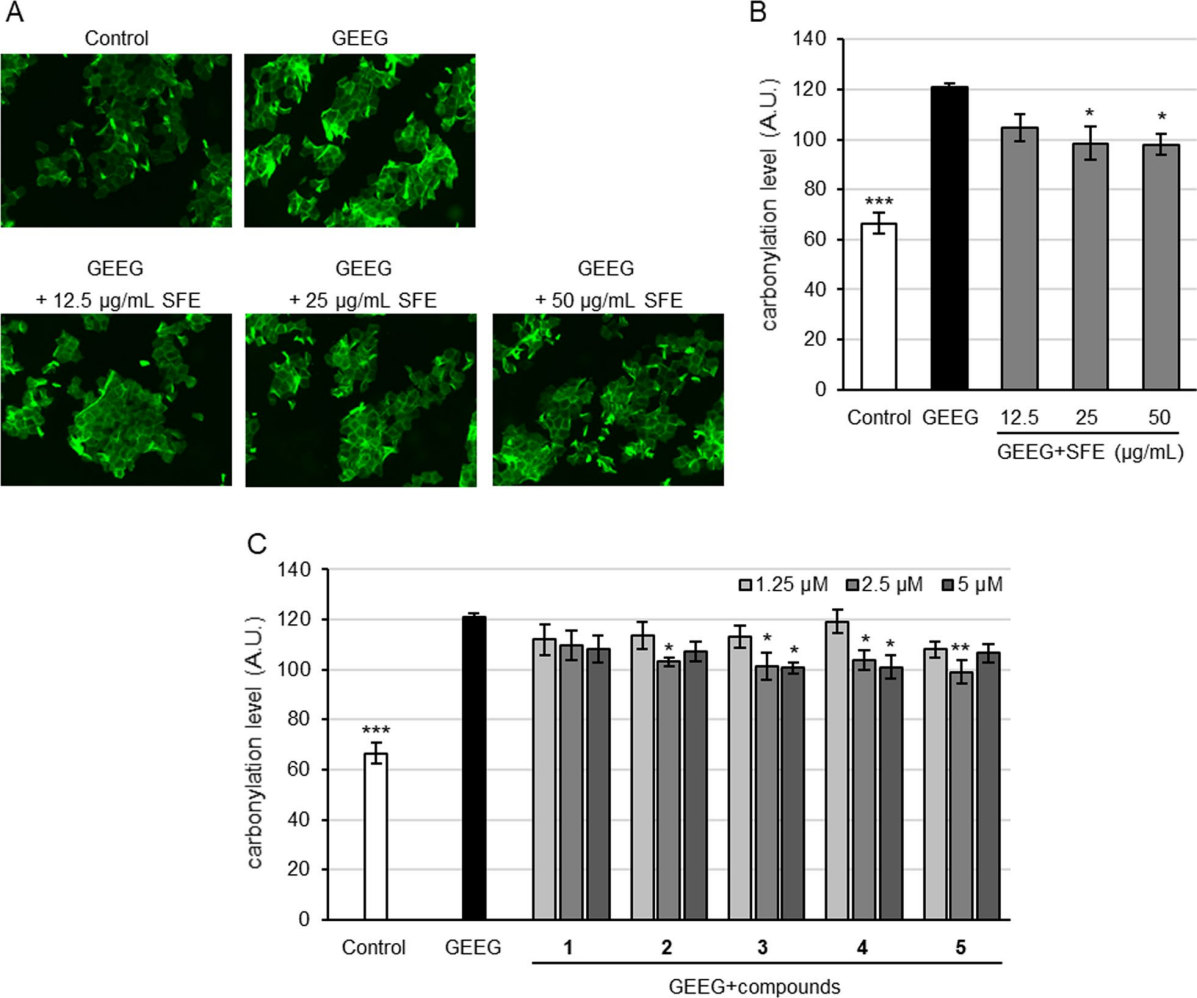
Inhibitory activity of SFE and compounds **1**–**5** against GEEG-induced protein carbonylation in SC. Tape-stripped SC was treated with SFE or components for 16 h at 37 °C. SC was stored in the absence or presence of GEEG for 24 h at 22 °C. Protein carbonylation in SC were detected by 5-FTSC. Inhibitory activity of SFE (**A**,** B**) and compounds **1**–**5** (**C**) against GEEG-induced protein carbonylation in SC and typical fluorescence image of protein carbonylation (**A**). Bars in B and C represent mean ± S.E., n = 3; *p < 0.05, **p < 0.01, ***p < 0.001 vs. GEEG-treated control.

To examine the activity of SFE and the components against actual air pollutants, inhibition of GEEG-induced protein carbonylation in SC was also evaluated. As shown in Fig. 3A, protein carbonylation level in SC was increased by treatment with GEEG as that of NaClO, and the pre-treatment with SFE significantly (*p* < 0.05) inhibited GEEG-induced protein carbonylation in SC at the dose of 25 and 50 μg/mL. All the tested compounds, except carambolaside I (**1**), were also significantly (*p* < 0.05) active against the protein carbonylation in SC at the dose of 2.5 or 5 μM (Fig. 3C; Additional file 1: Table S3).

### Inhibition of inflammation related mRNA expression in PM-stressed NHEKs by SFE and carambolaside P (3)

**Fig. 4:**
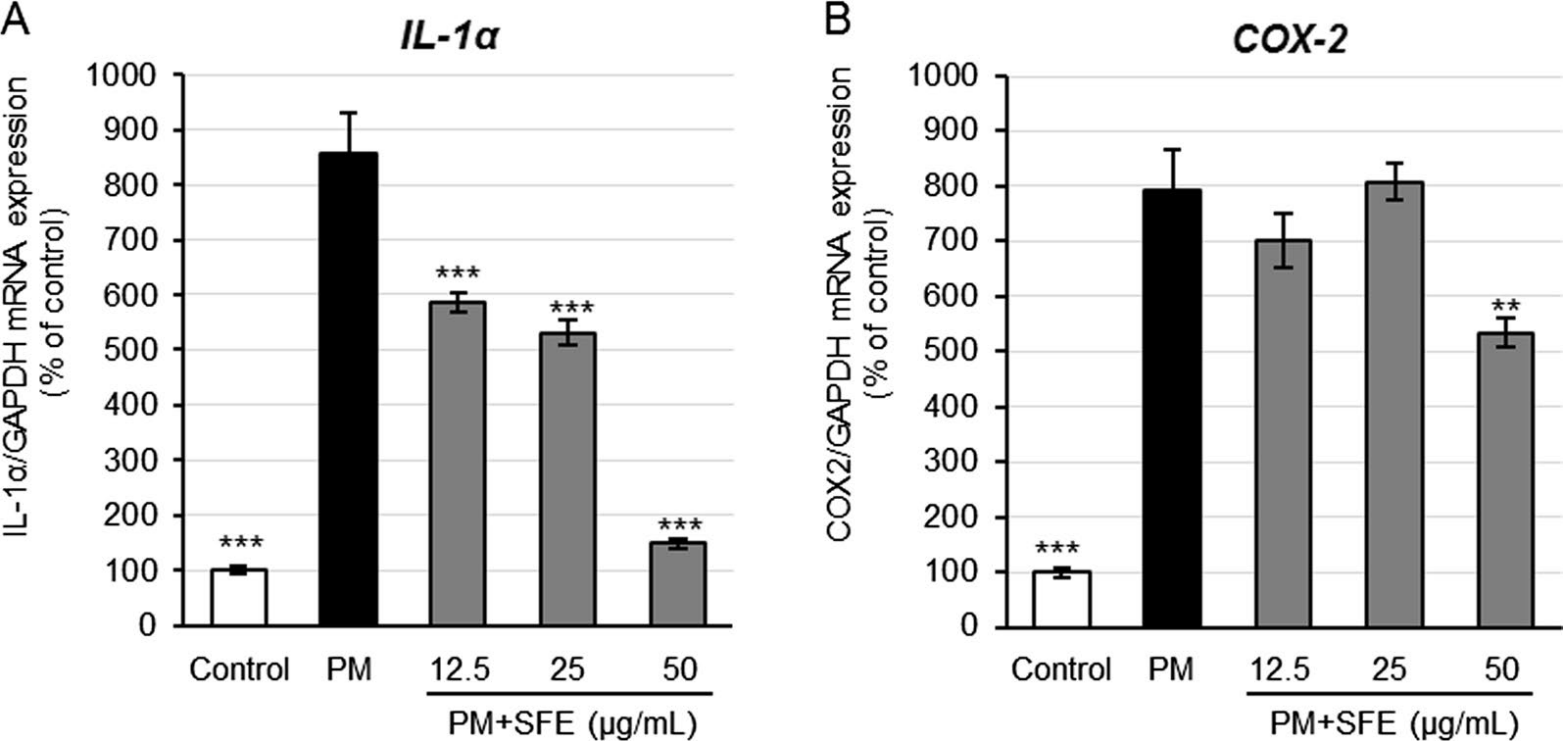
Inhibitory effects of SFE on IL-1α and COX-2 mRNA expression in PM-stressed keratinocytes. NHEKs were treated with SFE in the absence or presence of PM for 6 h at 37 °C. Each mRNA expression in the cells was examined by qRT-PCR. Inhibitory effects of SFE against PM-induced IL-1α (**A**) and COX-2 (**B**) mRNA expression in the cells. Bars represent mean ± S.E., n = 3; **p < 0.01, ***p < 0.001 vs. PM-treated control.

**Fig. 5:**
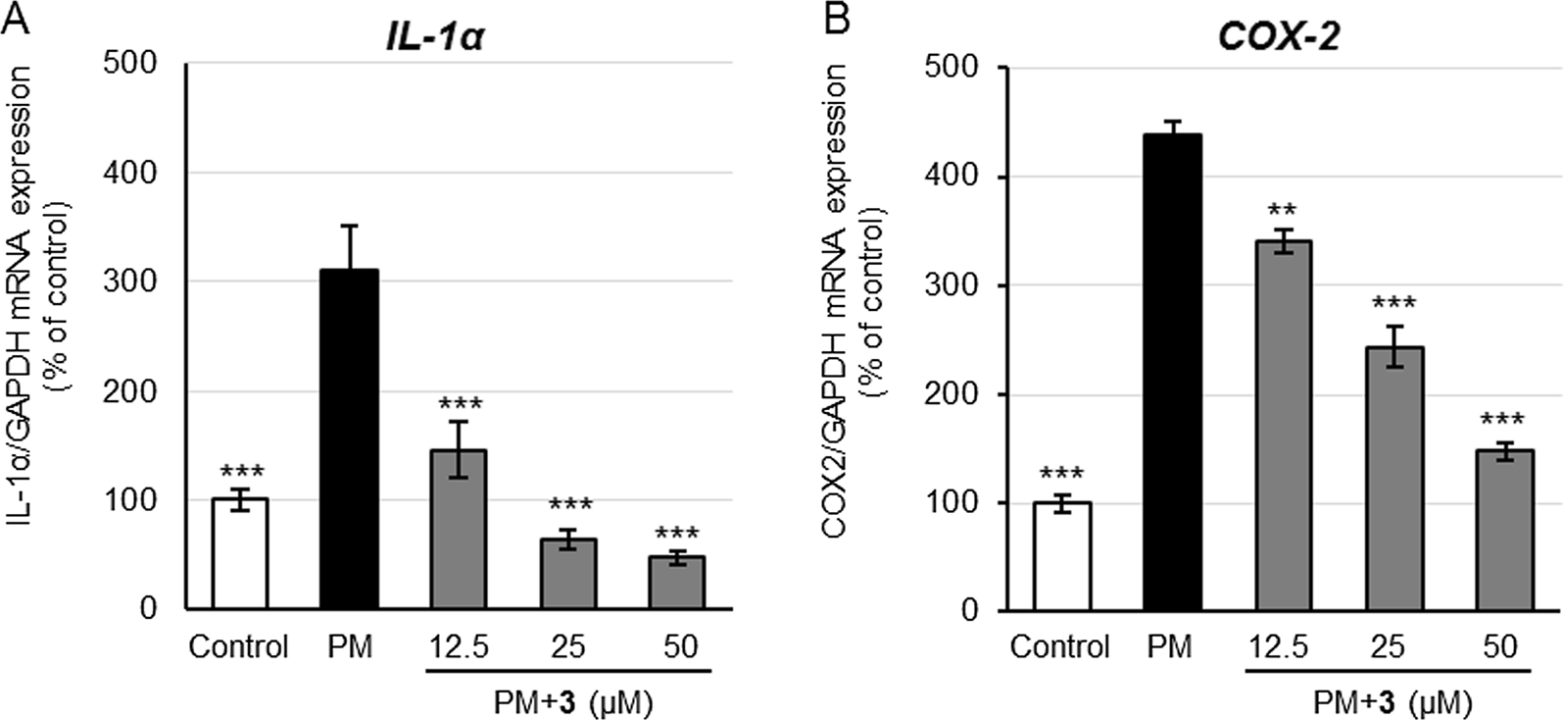
Inhibitory effects of compound **3** on IL-1α and COX-2 mRNA expression in PM-stressed keratinocytes. NHEKs were treated with compound **3** in the absence or presence of PM for 6 h at 37 °C. Each mRNA expression in the cells was examined by qRT-PCR. Inhibitory effects of compound **3** against PM-induced IL-1α (**A**) and COX-2 (**B**) mRNA expression in the cells. Bars indicate mean ± S.E., n = 3; **p < 0.01, ***p < 0.001 vs. PM-treated control.

To examine protective activity of SFE and components against air pollutant-induced skin diseases and premature skin aging, inhibitory effects of SFE and carambolaside P (**3**) on inflammation related IL-1α and COX-2 mRNA expression in PM-stressed NHEKs were evaluated by referring to the method of Ushio et al. [8] and Lee et al.[23]. SFE was shown to exhibit significant activity against IL-1α mRNA expression in a dose-dependent manner at concentrations ranging 12.5–50 μg/mL (Fig. 4A) and significantly inhibit COX-2 gene expression at the highest test concentration (50 μg/mL) (Fig. 4B) in PM-stressed NHEKs. In the evaluation, compound **3** displayed dose-dependent inhibition of gene expression of both IL-1α (Fig. 5A) and COX-2 (Fig. 5B) in the cells at the test concentrations (12.5–50 μM). These findings suggested that SFE and compound **3** were effective against PM-induced skin inflammation.

## Discussion

The flavonoid *C*-glycosides in star fruit mostly have d-fucose as the *C*-glycosylating moiety [19–21], which is distinctive from other dietary flavonoids. Besides, most star fruit flavonoid *C*-fucosides are featured with the dihydrochalcone phloretin as their aglycone, of which many are 3′-*C*-diglycosides, containing another d-fucose or a d-glucose linked to the *C*-fucose moiety by an *O*-glycosidic bond, and some are still 6′-*O*-diglycosylated by l-(2-*O*-d-fucosyl)arabinose, as represented by compounds **1**–**3**. Other main *C*-glycosylated flavonoids in star fruit are apigenin 6-*C*-diglycosides including the fucosyl-bearing **4** [19]. With these unique structural characteristics, the star fruit flavonoid *C*-glycosides are highly attractive in discovery of new biologically active substances from dietary resources. Furthermore, star fruit flavonoid *C*-glycosides are highly hydrophilic owing to bearing multiple (2–4) sugar units and are expected to have good moisture-retention capacity and thus to prevent skin dryness when topically applied. Therefore, the flavonoid *C*-glycosides **1**–**5** were investigated for their protective activity against air pollutant-induced skin disorders.

In evaluation for the activity against protein carbonylation in oxidative-stressed SC using NaClO as an oxidant, SFE and all tested flavonoid *C*-glycosides showed the activity (Fig. 2). Among three phloretin *C*-fucosides, compound **3** provided better activity than compounds **1** and **2** (Fig. 2C), which is attributable to the additional phenolic OH group contributed by the *trans*-*p*-coumaroyl attached to the *C*-fucose moiety (Fig. 1) and compound **1** showed activity superior to that of **2**, suggesting that *O*-fucosylation of the 3′-*C*-fucosyl moiety slightly decreases the activity. Comparison of compound **4** with **5** hinted that the 6-*C*-fucosylated flavones have the activity inferior to that of their 6-*C*-glucosylated counterparts. In the investigation with GEEG as an actual air pollutant, we first examined the response of SC to the pollutant and demonstrated that protein carbonylation in SC could be induced by GEEG (Fig. 3A), which is attributable to the oxidizing action arising from the redox-active components nitrogen dioxide, sulfur dioxide, ozone, etc. in GEEG [24, 25]. In the subsequent anti-GEEG activity evaluation by pretreating SC with the test samples, we found that SFE was also active against GEEG-induced protein carbonylation in the SC (Fig. 3A, B) and compounds **1**–**5** showed an activity profile (Fig. 3C) generally consistent with that of their activity against NaClO-induced protein carbonylation, except that compound **2** displayed activity slightly superior to that of **1**, suggesting that the inhibitory effect on GEEG-induced protein carbonylation in SC was due to antioxidant capacity of the test extract and compounds. The subsequent experiments were carried out with compound **3** as a representative of the active flavonoid *C*-glycosides in SFE since this compound showed the best activity in inhibition of the protein carbonylation and the highest relative content in the UPLC-UV analysis.

When the barrier function of the skin is impaired and the skin is exposed to air pollutants, it is thought that the PM in air pollutants may enter the epidermis and induce inflammation in keratinocytes. Therefore, we next evaluated the activity against skin inflammation caused by air pollutants. PM, containing polycyclic aromatic hydrocarbons that activate AhR, is known to induce inflammation in keratinocytes. Although commercial diesel exhaust particle has been evaluated as PM sources, the dust collected from fans in automobile tunnels (tunnel dust, TD) with specified polycyclic aromatic hydrocarbons and heavy metal contents was obtained and used as the PM sample in our evaluation. The influence of PM has been evaluated according to particle size, such as PM_2.5_ and PM_10_. The TD, with particle size ranging from 4.7 to 54 µm, is considered to be equivalent to PM_10_. It has been reported that treatment of HaCaT cell line with commercial PM_10_ upregulates IL-1α mRNA expression and damages the cells [22]. In our evaluation, the PM suspension made from TD was also found to induce IL-1α and COX-2 gene expression in NHEKs and its concentration-dependent effect was observed in the range of 1–20 µg/mL (data not shown). In response to the stimulation with 20 µg/mL of TD, both SFE and compound **3** significantly suppressed expression of both the genes (Figs. 4 and 5), indicating they are active against PM-induced skin inflammation. It has been reported that the induction of AhR-mediated inflammation by PM is an action of ROS, and the results of present study also suggest that the effect of SFE and compound **3** is mediated by their antioxidant effect. Taken together, it was suggested that SFE and flavonoid *C*-glycosides prevent skin dryness by inhibiting SC protein carbonylation caused by exhaust gases, improve skin optical properties to maintain skin beautiful appearance, and inhibit skin inflammation caused by dryness and PM. Thus, they are potentially effective against skin inflammation and premature aging caused by both gaseous and PM components in air pollution.

## Conclusion

The present study showed that the extract and the main *C*-glycosylated flavonoid constituents (**1**–**5**) of star fruit can decrease the levels of protein carbonylation in oxidative- and air pollutant-stressed SC. The extract and carambolaside P (**3**) can also inhibit mRNA expression of the inflammation mediators IL-1α and COX-2 in PM-stressed keratinocytes. These results, in combination with their dietary origin, suggested that the star fruit extract and *C*-glycosylated flavonoid components are promising as safe and effective additives for development of cosmetic products for preventing air pollutant-induced skin diseases and premature aging.

## Materials and methods

### Star fruit extract and components

To prepare the extract, the fresh fruits of *Averrhoa carambola* L. (purchased at the Chang-Ban Vegetable Market, Chang-Xing Rd. Tianhe District, Guangzhou, China, in June of 2019) were sliced and dried by heating at 60 °C. The dry fruit powder (100 g) was extracted with 60% ethanol (1.5 L) under reflux for 2 h. The extraction mixture was filtered and the filtrate was condensed under vacuum to obtain *A. carambola* fruit extract (SFE, 30 g), which was stored in the dark at -30 °C until use.

The compound samples of carambolasides I, J, and P (**1**–**3**), carambolaflavone B (**4**), and isovitexin 2″-*O*-α-L-rhamnoside (**5**) were obtained in our previous investigations [19–21] and stored in the dark at -20 °C until use.

### UPLC-HRESIMS analysis of SFE

SFE was constituted to 100 μg/mL with 20% MeCN and the solution was filtered through a 0.45 μm microporous membrane and 8 μL of the filtrate was injected for the analysis. The analysis was conducted on a Waters UPLC-MS system consisting of an Acquity H-Class UPLC system and a Xevo-G2 Q-ToF MS/MS instrument. The UPLC was equipped with an Acquity UPLC HSS C18 column (1.8 μm, 150 × 2.1 mm). The mobile phase consisted of 0.1% formic acid in water (A) and MeCN containing 0.1% formic acid (B). The gradient elution program was as follows: 0 min, 20% B; 11.5 min, 40% B; 12 min, 100% B; 13 min, 100% B. The flow rate was 0.3 mL/min, the column temperature was 40 °C, and the detection wavelength was 280 nm. The MS analysis was performed in the negative ion mode. The optimized parameters were as follows: capillary voltage, 2 kV; sampling cone voltage, 40 V; extraction cone voltage, 4 V; source temperature, 120 °C; desolvation temperature, 450 °C; cone gas flow, 50 L/h; desolvation gas flow, 1000 L/h.

### Anti-protein Carbonylation Assay in NaClO- and GEEG-stressed SC

Protein carbonylation assay was carried out based on the methods reported by Iwai et al. [14]. Briefly, SC was collected from volunteer’s upper arm by tape-stripping. To evaluate the activity against oxidative stress, sodium hypochlorite (NaClO) (FUJIFILM Wako Pure Chemical Corporation, Tokyo, Japan) was used as an oxidant. The SC was treated with a test sample (SFE or components) in the absence or presence of NaClO (40 µM) at 37 °C. After 16 h treatment, the SC was washed 2 times with 0.1 µM sodium 2-morpholinoethane sulfonate (DOJINDO LABORATORIES, Kumamoto, Japan) buffer (pH 5.5) (MES-buffer). Protein carbonyls in SC were determined as described below. For evaluation of the anti-GEEG activity, the SC was treated with a test sample for 24 h at 37 °C. The treated SC was placed in a plastic bag, GEEG was put into the bag and sealed, and the SC in bag was stored at 22 °C for 24 h. After the SC was washed 2 times with MES-buffer, protein carbonyls in the SC were determined as described below.

### Detection of protein carbonyls in SC

Protein carbonyls in SC were determined using the methods reported by Fujita et al. [22]. Briefly, the SC was labeled with 20 µM fluorescein-5-thiosemicarbazide (5-FTSC, AnaSpec, Fremont, CA., USA) in MES-buffer for 1 h at 22 °C, and washed 3 times with PBS. The fluorescence staining image was captured by a BZ-X800 fluorescent microscope (Keyence, Osaka, Japan). The average fluorescence intensity of the area evaluated by image analysis was expressed as the carbonylation level of SC protein in arbitrary units. The inhibitory effect of test sample on carbonylation was expressed as percentages of decrease from the carbonylation level of sample untreated control SC.

### Cell culture

Normal human epidermis keratinocytes (NHEKs) were purchased from KURABO Industries Ltd. (Osaka, Japan) and cultured in serum-free keratinocyte basic medium (KBM, trade names: HuMedia-KB2) containing human epidermal growth factor, insulin, hydrocortisone, gentamycin/amphotericin B, and bovine pituitary extract (keratinocyte growth medium, KGM, trade names: HuMedia-KG2) (KURABO Industries Ltd., Osaka, Japan) at 37 °C in an atmosphere consisting of 5% CO_2_.

### Air pollution induced-gene expression

In this study, tunnel dust (TD) was used as particles matter (PM) sample. TD was purchased from National Metrology Institute of Japan (NMIJ, Ibaraki, Japan) of National Institute of Advanced Industrial Science and Technology (AIST, Tokyo, Japan) as certified reference material (NMIJ CRM 7308-a No. A027). PM suspension was freshly prepared by re-suspending TD particles (50 mg/mL stock solution in PBS) in culture medium at the final concentration of 20 µg/mL, followed by incubation (10 min) under ultrasonication in an ultrasonic water bath. NHEKs were collected by trypsin treatment and diluted with KGM to create a concentration of 2 × 10^5^ cells/mL, and 2 mL of the suspended cells were seeded onto a 6-well plate for overnight incubation. After incubation, 2 mL of KGM was replaced by 2 mL of KBM for 24 h incubation. The medium was removed and 2 mL of PM and a test sample dissolved in KBM was added to the plate. Following 6 h of incubation, total RNA was isolated using the standard method.

### Quantitative real-time RT-PCR

Total RNA was isolated from NHEKs using ISOGEN II (Nippon Gene, Tokyo, Japan) in accordance with a standard operating method, and cDNAs were synthesized with PrimeScript RT Master Mix using TaKaRa PCR Thermal Cycler Dice Touch (TaKaRa Bio, Siga, Japan). Real-time RT-PCR for IL-1α, COX-2, and GAPDH was performed with TB Green Fast qPCR Mix using Thermal Cycler Dice Real Time System III (TaKaRa Bio, Siga, Japan), and the primer sets were purchased from Takara Bio. The IL-1α and COX-2 mRNA expression was normalized to glyceraldehyde-3-phosphate dehydrogenase (GAPDH) expression and expressed as percentages against the expression in test sample untreated control cells.

### Statistical analysis

All data are expressed as the mean ± standard error of the mean (S.E.) of at least three independent determinations for each experiment. A multiple comparison between individual treatments and stimulated controls were performed using the Dunnett test. A *p*-value of < 0.05 was considered statistically significant. As no drugs or agents are available for the treatment or prevention of air pollutant-induced skin disorders at present, all activity evaluations were carried out without any positive controls.

## Supplementary Information

**Additional file 1: Fig. S1.** UPLC-UV chromatogram of SFE. **Fig. S2.** UPLC-MS chromatogram of SFE. **Table S1.** Detected flavonoid C-gylycosides in SFE. **Table S2.** Activity against NaClO-induced protein carbonylation in SC. **Table S3.** Activity against GEEG-induced protein carbonylation in SC.
