# Supplementary material for: Star fruit extract and C-glycosylated flavonoid components have potential to prevent air pollutant-induced skin inflammation and premature aging
Source: Nat Prod Bioprospect. 2022 Apr 1;12(1):13. doi: 10.1007/s13659-022-00336-1 (PMC8971273; doi:10.1007/s13659-022-00336-1)
Supplement: Supplementary file 1 — Additional file 1: Fig. S1. UPLC-UV chromatogram of SFE. Fig. S2. UPLC-MS chromatogram of SFE. Table S1. Detected flavonoid C-gylycosides in SFE. Table S2. Activity against NaClO-induced protein carbonylation in SC. Table S3. Activity against GEEG-induced protein carbonylation in SC. [file 13659_2022_336_MOESM1_ESM.pdf]

Supplementary Information

Star Fruit Extract and *C*-Glycosylated Flavonoid Components Have Potential to Prevent Air Pollutant-induced Skin Inflammation and Premature Aging

Ping Wu, Hiroyasu Iwahashi,* Haihui Xie, Ying Wang, Yanyang Zhou, Akinori Kiso, Yoshihito Kawashima, and Xiaoyi Wei*

*Corresponding authors: E-mail: h-iwahashi@maruzenpcy.co.jp (HI); wxy@scbg.ac.cn (XW).

Contents

1. UPLC-HRESIMS Analysis of Star Fruit Extract (SFE)

Fig. S1 UPLC-UV chromatogram of SFE 2

Fig. S2 UPLC-MS chromatogram of SFE 2

Table S1 Detected flavonoid *C*-gylycosides in SFE ………………………………3

2. Activity of SFE and Compounds 1–5 against NaClO- and GEEG-induced Protein Carbonylation in SC

Table S2. Activity against NaClO-induced protein carbonylation in SC …………...4

Table S3. Activity against GEEG-induced protein carbonylation in SC ……………5

1. UPLC-HRESIMS Analysis of Star Fruit Extract (SFE)

<UV 280 nm クロマトグラム>

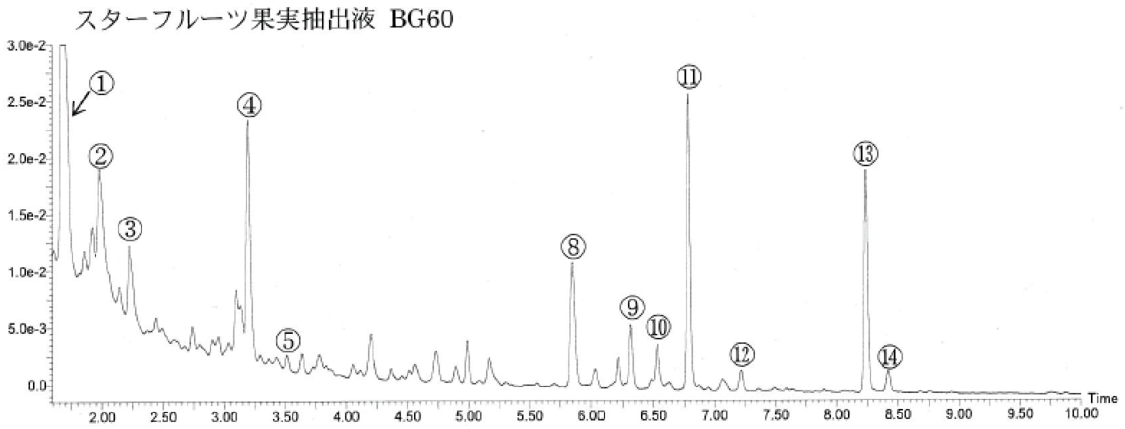

Fig. S1 UPLC-UV chromatogram of SFE (detected at 280 nm).

<MS クロマトグラム (*m/z* : 500-1000)、ESI negative>

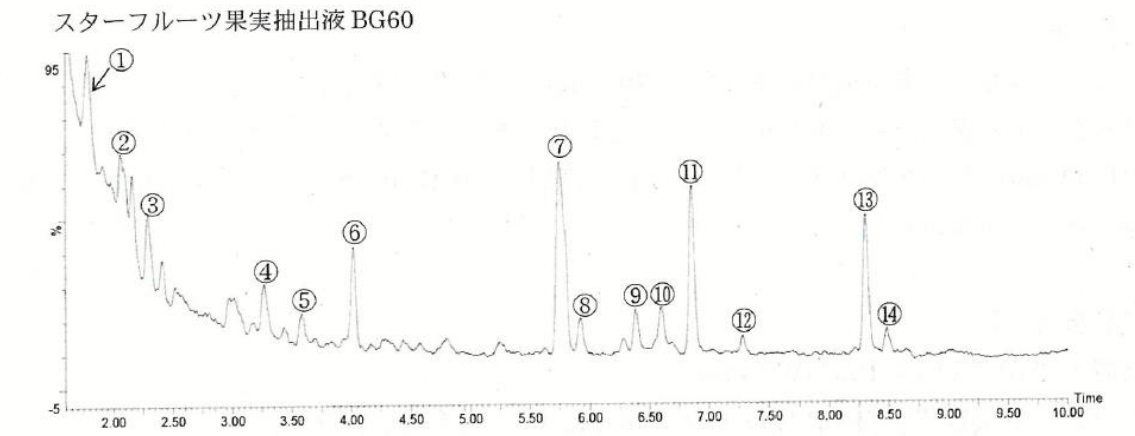

Fig. S2 UPLC-MS chromatogram of SFE (negative ion mode, *m/z* 500–1000).

Table S1 Detected flavonoid *C*-gylycosides in SFE by UPLC-MS analysis

peak No.[a]	RT (min)	formula	$[M-H]^-$ (*m/z*)	calcd (*m/z*)	identification
1 [b]	1.78	$C_{30}H_{25}O_{12}$	577.1328	577.1346	-
2	2.06	$C_{15}H_{13}O_5$	289.0705	289.0712	epicatechin
3 [c]	2.29	$C_{27}H_{29}O_{14}$	577.1565	577.1557	isovitexin 2″-α-L-Rha (**5**)
4 [b]	3.28	$C_{15}H_{19}O_4$	263.1275	263.1283	-
5	3.59	$C_{32}H_{41}O_{17}$	697.2356	697.2344	carambolaside Ia
6 [b]	4.02	$C_{27}H_{41}O_{12}$	557.2539	557.2598	-
9	6.38	$C_{47}H_{57}O_{23}$	989.3306	989.3291	carambolaside O
10	6.60	$C_{41}H_{47}O_{19}$	843.2706	843.2712	carambolaside Q
11 [c]	6.85	$C_{47}H_{57}O_{23}$	989.3311	989.3291	carambolaside P (**3**)
12	7.28	$C_{47}H_{57}O_{22}$	973.3400	973.3341	isomer of carambolaside J
13 [c]	8.30	$C_{47}H_{57}O_{22}$	973.3336	973.3341	carambolaside J (**2**)
14 [c]	8.48	$C_{41}H_{47}O_{18}$	827.2771	827.2762	carambolaside I (**1**)

[a] Peak 8 in Fig. S1 and peak 7 in Fig. S2 not listed as they are obvious non-flavonoid impurities as suggested by their discrepancies between UV- and MS-detected UPLC.

[b] Not characterized.

[c] Confirmed by comparison with the reference compounds (**1**–**5**) isolated from star fruits.

2. Activity of SFE and Compounds 1–5 against NaClO- and GEEG-induced Protein Carbonylation in SC

Table S2. Inhibitory activity of SFE and compounds **1–5** against NaClO-induced protein carbonylation in SC

induce	sample	conc.	Inhibitory rate (%)		IC_{50}
NaClO(-)	Normal	0	100.0 ± 6.7	***	
NaClO(+)	Control	0	0.0 ± 6.6		
	SFE	12.5	9.0 ± 3.7		
	(μg/mL)	25	13.6 ± 6.7		43.2 μg/mL
		50	62.6 ± 17.1	**	
	Compound **1**	1.25	29.3 ± 1.7	*	
	(μM)	2.5	35.6 ± 7.1	**	3.5 μM
		5	88.5 ± 3.9	***	
	Compound **2**	1.25	31.7 ± 7.3	*	
	(μM)	2.5	23.8 ± 9.2		5.0 μM
		5	51.2 ± 9.5	***	
	Compound **3**	1.25	38.3 ± 5.2	**	
	(μM)	2.5	93.4 ± 3.5	***	1.5 μM
		5	111.8 ± 0.8	***	
	Compound **4**	1.25	18.6 ± 5.4		
	(μM)	2.5	20.9 ± 4.4		>5.0 μM
		5	41.4 ± 6.1	**	
	Compound **5**	1.25	21.4 ± 10.8		
	(μM)	2.5	47.7 ± 4.8	***	2.6 μM
		5	88.2 ± 12.1	***	

Mean ± SE, n =3; * $P < 0.05$; ** $P < 0.01$; *** $P < 0.001$.

Table S3. Inhibitory activity of SFE and compounds **1–5** against GEEG-induced protein carbonylation in SC

induce	sample	conc.	Inhibitory rate (%)	
GEEG(-)	Normal	0	100.0 ± 7.7	***
GEEG(+)	Control	0	0.0 ± 3.2	
	SFE	12.5	9.0 ± 3.7	
	(µg/mL)	25	13.6 ± 6.7	*
		50	62.6 ± 17.1	*
	Compound **1**	1.25	16.0 ± 11.3	
	(µM)	2.5	20.9 ± 10.9	
		5	22.9 ± 9.9	
	Compound **2**	1.25	29.7 ± 10.2	
	(µM)	2.5	32.6 ± 2.8	*
		5	25.0 ± 7.6	
	Compound **3**	1.25	14.4 ± 8.3	
	(µM)	2.5	35.7 ± 9.9	*
		5	37.0 ± 4.0	*
	Compound **4**	1.25	2.9 ± 8.9	
	(µM)	2.5	31.1 ± 7.1	*
		5	36.4 ± 8.9	*
	Compound **5**	1.25	23.6 ± 5.9	
	(µM)	2.5	40.1 ± 8.8	**
		5	26.2 ± 6.9	

Mean ± SE, n =3; * $P < 0.05$, ** $P < 0.01$, *** $P < 0.001$.
